# Scalability Metrics and Effort Requirements for a Long-Acting Injectable Antiretroviral Treatment Program

**DOI:** 10.1093/ofid/ofag116

**Published:** 2026-03-04

**Authors:** Joshua P Havens, Jennifer O’Neill, Maureen Kubat, Shawnalyn W Sunagawa, Jennifer M Davis, Nada Fadul, Joshua Lechner, Sara H Bares

**Affiliations:** Division of Infectious Diseases, University of Nebraska Medical Center, College of Medicine, Omaha, Nebraska, USA; Department of Pharmacy Practice, University of Nebraska Medical Center, College of Pharmacy, Omaha, Nebraska, USA; Division of Infectious Diseases, University of Nebraska Medical Center, College of Medicine, Omaha, Nebraska, USA; Division of Infectious Diseases, University of Nebraska Medical Center, College of Medicine, Omaha, Nebraska, USA; Department of Pharmacy Practice, University of Nebraska Medical Center, College of Pharmacy, Omaha, Nebraska, USA; Division of Infectious Diseases, University of Nebraska Medical Center, College of Medicine, Omaha, Nebraska, USA; Division of Infectious Diseases, University of Nebraska Medical Center, College of Medicine, Omaha, Nebraska, USA; Nebraska Medicine, Omaha, Nebraska, USA; Division of Infectious Diseases, University of Nebraska Medical Center, College of Medicine, Omaha, Nebraska, USA

**Keywords:** cabotegravir/rilpivirine, implementation, long-acting injectable ART

## Abstract

Implementation of a long-acting injectable antiretroviral treatment program requires substantial multidisciplinary effort, particularly for program coordination, coverage/billing, and patient support/retention. As our program scaled to 113 patients over 2.5 years, a total of 2.25 full-time equivalents were required. Despite operational demands, clinical outcomes were favorable, supporting real-world feasibility and scalability.

Long-acting injectable (LAI) antiretroviral therapy (ART) with cabotegravir and rilpivirine (CAB/RPV), introduced in 2021, represents a paradigm shift in human immunodeficiency virus-1 management [1, 2]. Long-acting injectable ART offers patient-centered benefits including eliminating the burden of daily oral medication [3–5], reducing potential stigma [6, 7], and improving treatment satisfaction and quality of life [3–5, 8]. Efficacy and LAI preference are established in both clinical trial [3–5, 9] and real-world [10–13] settings across various populations including treatment naive [4] and experienced [4, 5, 9], as well as cohorts of patients who struggle with adherence and viremia [14–16].

However, its transition to widespread real-world use has been slowed by significant operational and logistical barriers [17–19]. Early implementation studies consistently highlight challenges with substantial staffing requirements [20, 21], complex insurance/billing [17, 22–25], and the need for new clinical workflows [21, 22, 26]. There remains a scarcity of longitudinal LAI program scale-up data describing staffing models, evolving effort requirements, and scalability metrics as a program matures. Understanding these dynamics is critical for healthcare systems to forecast resource needs, provide equitable LAI access, develop sustainable LAI programs, and adapt to the evolving HIV treatment landscape [26].

This retrospective study addresses the knowledge gap by describing the staff effort, resource allocation, and scalability metrics of a real-world LAI CAB/RPV program over a 2.5-year period at the Specialty Care Center (SCC), the University of Nebraska Medical Center HIV clinic.

## METHODS

### Study Design and Setting

This was a retrospective, time-allocation cohort study conducted from May 2022 to December 2024 at the SCC. The SCC is a Ryan White–funded HIV clinic serving ∼1300 people with HIV (PWH). The SCC's LAI program was developed based on the clinic's experience as a clinical trial site [3–5, 27, 28] and primarily utilizes a buy-and-bill mechanism [29] (98% of LAI injections) for medication procurement and administration (Supplementary Figure 1).

### Participant Population

All adult (≥19 years old in Nebraska) PWH who received LAI ART with CAB/RPV for HIV treatment at the SCC during the study period were included in the analysis.

### Data Collection and Outcomes

The primary outcome was the estimated staff effort required to support the SCC LAI program, measured in full-time equivalent (FTE) units. FTE estimates were calculated based on direct observation, staff interviews, and review of departmental effort reporting records for time spent on LAI-specific activities. This effort was categorized by staff role (eg, LAI program coordinator, provider, registered nurse) and by specific task categories (eg, LAI program coordination, patient outreach, coverage/prior authorizations) and then extrapolated to dedicated FTE by role. Scalability metrics (patients/FTE and injections/FTE) were calculated annually to assess changes in program efficiency.

Secondary outcomes included injection adherence and clinical outcomes. These were defined as follows: 1() delayed injection, an injection administered >7 days past the target date with subsequent use of oral bridging medication; (2) missed injection, an injection administered >7 days past the target date without oral bridging; (3) virologic blip, a single detectable HIV RNA >50 copies/mL with a subsequent return to <50 copies/mL on repeat check; and (4) virologic failure, 2 consecutive HIV RNA measurements >200 copies/mL.

### Statistical Analysis

Descriptive statistics (medians, interquartile ranges [IQRs], frequencies, and percentages) were used to summarize participant characteristics, FTE allocation, and clinical outcomes. Year-over-year percentage changes assessed trends in scalability metrics.

## RESULTS

### Cohort and Program Growth

From May 2022 to December 2024, a total of 113 participants received LAI ART. The cohort had a median age of 44 years (IQR, 35–58), was predominantly male (79%), White (78%), and included 32% residing in rural areas (Supplementary Table 1). The SCC LAI program experienced rapid and sustained growth throughout the study period. The annual number of injections administered increased from 68 in the second half of 2022 to 301 in 2023 and 515 in 2024, representing a 657% increase from program launch through the end of the study period (Table 1).

**Table 1.** LAI Program Scalability Metrics, Injection, and Clinical Outcomes

LAI Scalability Metrics, n (YoY Δ)	2022	2023	2024
Total patients	27	62 (129%)	113 (82%)
Total injections	68	301 (343%)	515 (71%)
New patients	…	35	51
Dedicated FTE	1.0	1.5	2.25
Patients/FTE	27	41 (52%)	50 (22%)
Injections/FTE	68	201 (196%)	229 (14%)
New patients/FTE	…	23	26 (9%)
**Injection Outcomes**	**Injections, n (%)**	**Comments**	
Delayed	8 (0.9)	All received <1 m of oral bridging	
Missed	3 (0.3)	Median (range) past injection window: 5 d (1–7)	
**Clinical Outcomes**	**Participants, n (%)**	**Comments**	
Virologic blips^a^	15 (13.3)	Total: 18; median (range) HIV RNA, 78 (51–489)	
Virologic failure	0 (0.0)	…	
CAB/RPV Discontinuations	3 (2.7)	Reasons: LLV, HBV, patient choice (n = 1 for each)	

Abbreviations: BMI, body mass index; CAB/RPV, cabotegravir/rilpivirine; FTE, full-time equivalent; HBV, hepatitis B; LAI, long-acting injectable; LLV, low-level viremia (HIV RNA >50 to <200 copies/mL); YoY Δ, year-over-year change.

^a^Two participants (11%) were receiving CAB/RPV monthly and had been viremia at initiation of CAB/RPV; 7 participants (39%) had a BMI >30 kg/m2 of which 4 participants (57%) received CAB/RPV every 2 m via administration with a 1.5″ needle.

### Staffing Effort and Allocation

**Figure 1.**
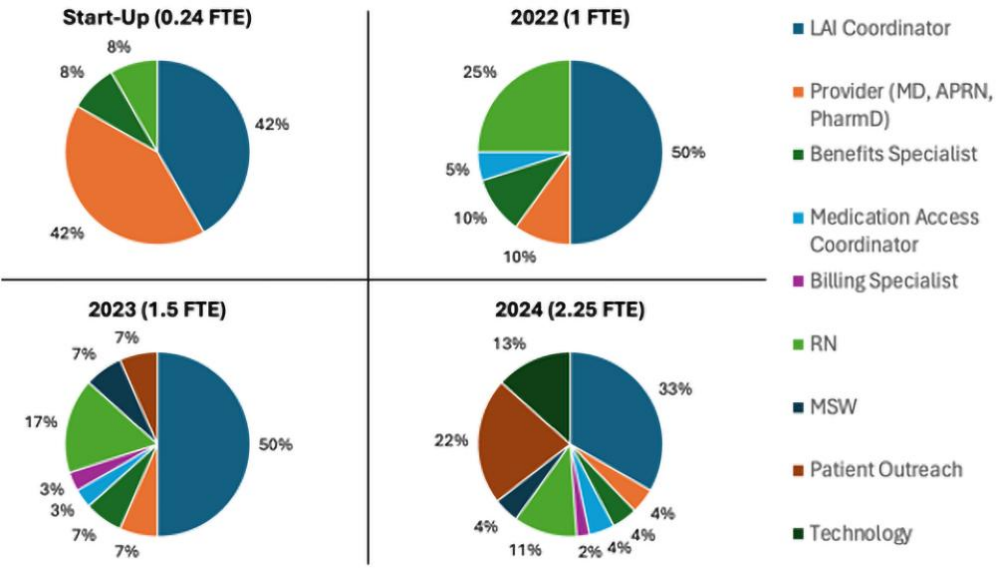
Dedicated full-time equivalent (FTE) for long-acting injectable (LAI) injectable cabotegravir/rilpivirine program. MD, medical doctor; APRN, advanced practice registered nurse; PharmD, pharmacist; RN, registered nurse; MSW, medical social worker; Technology, informatics/software development.

Prior to SCC LAI program launch, the initial start-up period required an estimated 0.24 FTE to manage protocol development, workflow processes, and LAI access/ordering logistics. After SCC LAI program initiation, the total dedicated staff effort increased from 1.0 FTE in 2022 to 2.25 FTE in 2024 with effort distribution across roles evolving as the program scaled (Figure 1).

The LAI program coordinator remained the largest single component, comprising 33%–50% of the total FTE each year. This role managed all LAI program aspects, including coordinating efforts, in-depth benefits/cost analyses, staff training, previsit planning, patient care/education, insurance claims review, patient retention, and insurance change coordination. The coordinator also drove program growth by leading data collection, problem-solving, collaborating with ancillary sites to expand LAI ART availability, and partnering with information technology infrastructure teams.

Proportional effort from clinical providers decreased substantially from 42% in 2022 to 4% in 2024 as the program matured. Administrative effort shifted over time with the addition of supportive roles: medication access coordinator (coverage, prior authorization, and patients savings program coordination), benefits/billing specialists, patient outreach staff, and technology staff (informatics/workflow build) (Figure 1).

Analysis of cumulative time spent on specific tasks confirmed program maturity, revealing a shift in LAI program focus toward workflow efficiencies such as patient outreach/tracking and technology development. Additionally, considerable time and effort were spent iterating and evolving the SCC LAI program (eg, team meetings, protocol adjustments, workflow optimization, scalability planning, etc.) (Supplementary Figure 2).

### Scalability and Efficiency

Despite increasing administrative effort, the SCC LAI program's overall efficiency improved. The number of patients managed (27–50) and injections (68–229) per dedicated FTE increased steadily from 2022 to 2024. This demonstrates a positive scalability trend, suggesting the program developed streamlined, effective workflows as it matured and expanded (Table 1).

### Clinical and Adherence Outcomes

Among the 113 participants, there were 8 instances of delayed injections (all managed with oral bridging) and 3 missed injections. Fifteen participants (13.3%) experienced a total of 18 virologic blips, with a median HIV RNA of 78 copies/mL (range: 51–489). Critically, no instances of virologic failure were observed throughout the study period. The rate of discontinuation was low, with only 3 participants (2.7%) stopping LAI CAB/RPV (low-level viremia [HIV RNA >50 to <200 copies/mL], n = 1; hepatitis B diagnosis, n = 1; patient choice, n = 1) (Table 1).

## DISCUSSION

This study provides longitudinal, real-world insights into implementing and scaling an LAI ART HIV treatment program. Effort dedicated to the LAI program increased substantially from 0.24 FTE at start-up to 2.25 FTE over 2.5 years. Our experience confirms that successful expansion requires substantial staff effort, a dynamic multidisciplinary approach, and a program champion to coordinate the complex administrative and logistical needs unique to LAI care. These elements were essential for balancing rapid scale-up with optimal clinical outcomes (ie, on-time injections and sustained virologic suppression).

We identified 2 critical operational elements: (1) a central coordinator and (2) proactive administrative bottleneck management. The required 0.33–0.5 FTE for the LAI program coordinator, consistent with early implementation studies [20], underscores the need for intensive management to navigate multilevel implementation complexities. Crucially, as the program matured, the effort focus shifted from coverage/billing to patient-centric interventions and technology to optimize LAI clinical workflows. We attribute this shift to the development of efficient workflows and building custom electronic health record technology (effort: ∼60 days; Compass Rose; Epic) that reduced the time traditionally required for administrative processes as staff gained LAI experience. This efficiency allowed the team to redirect effort toward patient outreach/follow-up and ensuring engagement in care, uninterrupted insurance/grant coverage (ie, mitigating coverage changes), and virologic suppression. While other access models (eg, clear- and white-bagging) typically shift coverage and billing aspects to a pharmacy partner, our buy-and-bill model intentionally takes on these logistic challenges to maintain programmatic control to ensure continued medication access. Consequently, the primary rate-limiting step to scale an LAI program, particularly within a buy-and-bill model, is optimizing patient workflow efficiency rather than initial logistical hurdles noted by other studies [22, 26, 30]. Thus, initial staffing models must be strategically designed to adapt to the changing administrative support and resource allocation needs of a mature program.

Our LAI program achieved marked efficiency improvement, nearly doubling patients managed per FTE over 2.5 years, likely due to our specialized multidisciplinary staffing model. Pairing dedicated, hybridized staff (clinical and implementation knowledge) with collaborative institutional departments (medication access, pharmacy, billing) effectively reduces friction and prevents administrative bottlenecks, allowing staff to operate at a higher capacity and focus on improving efficiency and patient outcomes.

We found clinical provider effort in day-to-day LAI program activity decreased from 42% to 4% over our study period. Despite this decrease in clinician effort with program maturity, our staffing model is validated by its exceptional clinical success with no virologic failures and a low discontinuation rate (2.7%). These findings reinforce that intensive coordination and robust infrastructure support are essential components of effective HIV LAI care, not merely operational costs. Investing in staff and technology to overcome real-world barriers (eg, patient outreach, insurance coverage, etc.) directly mitigates LAI ART non-adherence. Thus, preventing missed injections and virologic failure frames the FTE investment as a prerequisite for realizing the full clinical promise of LAI ART. While provider effort may fluctuate with episodic needs, it does not fully taper over time. Instead, a small but consistent effort is maintained by a limited number of providers who regularly provide clinical guidance as questions arise for assessing LAI eligibility, protocol updates, and complex case management.

This study has several limitations. As a single-center study conducted in an academic setting with a specific “buy-and-bill” access model and for HIV treatment, the findings may not be directly generalizable to all access models (eg, “white- or clear-bagging”), clinical settings (eg, HIV pre-exposure prophylaxis [PrEP] or other non-HIV LAI programs), or clinics with different demographic and payer mix proportions. Furthermore, the FTE data are based on systematic time-allocation estimates rather than direct time-motion tracking, which could introduce a degree of measurement error. Lastly, a portion of FTE (∼0.2) for the LAI program was funded by an industry-sponsored study that started in 2024 (IM-CAPABLE; NCT06451341).

## CONCLUSION

Successful implementation and scaling of an LAI program is operationally demanding, is resource intensive, and requires careful planning. Our findings provide a framework and model for other LAI programs to follow. Adequate, proactive investment in a multidisciplinary team is critical to overcoming logistical barriers and realizing the full potential of LAIs alongside HIV care innovation.

## Supplementary Material

ofag116_Supplementary_Data

## Supplementary Data

Supplementary materials are available at *Open Forum Infectious Diseases* online. Consisting of data provided by the authors to benefit the reader, the posted materials are not copyedited and are the sole responsibility of the authors, so questions or comments should be addressed to the corresponding author.
