# Supplementary material for: Scalability Metrics and Effort Requirements for a Long-Acting Injectable Antiretroviral Treatment Program
Source: Open Forum Infect Dis. 2026 Mar 4;13(3):ofag116. doi: 10.1093/ofid/ofag116 (PMC12981548; doi:10.1093/ofid/ofag116)
Supplement: ofag116_Supplementary_Data [file ofag116_supplementary_data.zip › CAB.RPV_scalability_Supplementary Table.docx]

**Supplementary Table 1. Participant Characteristics (2022-2024)**

| **Characteristic** | **N=113** |
| --- | --- |
| Age, median (IQR), years | 44 (35, 58) |
| Race, n (%)  White  Black  Other | 88 (78)  21 (18)  4 (4) |
| Ethnicity, n (%)  Hispanic  Not Hispanic | 15 (13)  98 (87) |
| Gender, n (%)  Male  Female  Transgender/Non-Binary | 90 (79)  19 (17)  4 (4) |
| Residence, n (%)  Urban  Rural | 77 (68)  36 (32) |
| Insurance Coverage, n (%)  Commercial  Medicare  Medicaid  Federal/Veterans Affair | 75 (66)  17 (15)  17 (15)  4 (4) |
| Social Deprivation Index, median (IQR) | 50 (19, 73) |
| BMI, n (%)  18-24.9 kg/m^2^  25-29.9 kg/m^2^  30-34.9 kg/m^2^  35-39.9 kg/m^2^  >40 kg/m^2^ | 33 (29.2)  39 (34.5)  25 (22.1)  11 (9.8)  5 (4.4) |
| CAB/RPV Dosing Formulation, n (%)  Monthly (400mg/600mg)^a^  Every 2 Months (600mg/900mg) | 2 (1.8)  111 (98.2) |

^a^Both participants were viremic at CAB/RPV initiation.

Definitions: IQR, interquartile range.
