# Supplementary figures and images for: Scalability Metrics and Effort Requirements for a Long-Acting Injectable Antiretroviral Treatment Program
Source: Open Forum Infect Dis. 2026 Mar 4;13(3):ofag116. doi: 10.1093/ofid/ofag116 (PMC12981548; doi:10.1093/ofid/ofag116)

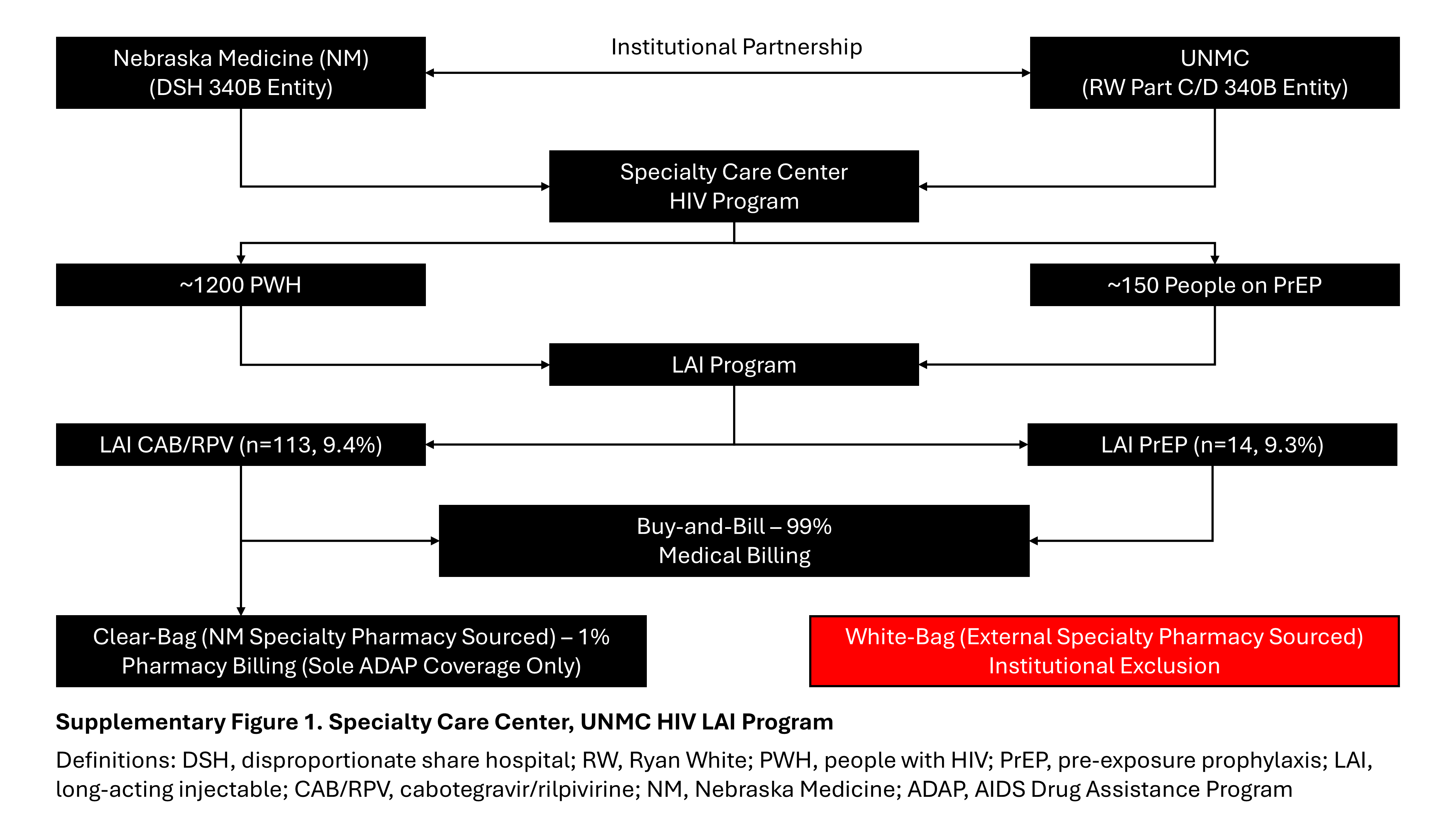

Supplement: ofag116_Supplementary_Data [file ofag116_supplementary_data.zip › HAVENS-SCALABILITY-SUPPFIG1.tif]

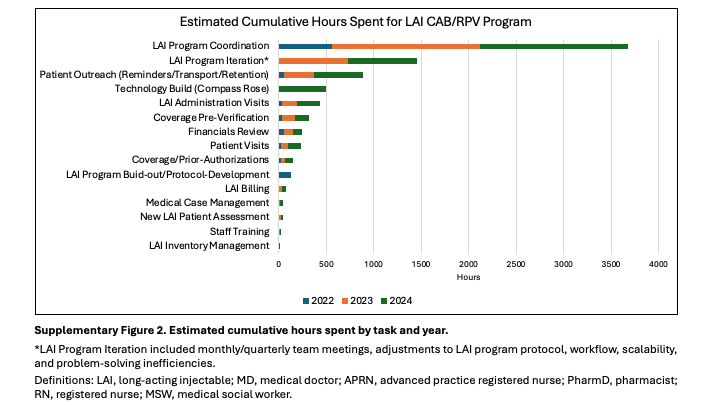

Supplement: ofag116_Supplementary_Data [file ofag116_supplementary_data.zip › HAVENS-SCALABILITY-SUPPFIG2.tiff]
